# Evaluation of decision-making capacity in patients with dementia: challenges and recommendations from a secondary analysis of qualitative interviews

**DOI:** 10.1186/s12910-020-00498-y

**Published:** 2020-07-06

**Authors:** Christopher Poppe, Bernice S. Elger, Tenzin Wangmo, Manuel Trachsel

**Affiliations:** 1 Institute for Biomedical Ethics, University of Basel, Basel, Switzerland; 2 Center for Legal Medicine, University of Geneva, Geneva, Switzerland; 3 Institute of Biomedical Ethics and History of Medicine, University of Zurich, Winterthurerstrasse 30, CH-8006 Zürich, Switzerland

**Keywords:** Decision-making capacity, Competence, Dementia, Informed consent, Ethics, Autonomy

## Abstract

**Background:**

Evaluation of decision-making capacity to consent to medical treatment has proved to be difficult in patients with dementia. Studies showed that physicians are often insufficiently trained in the evaluation of decision-making capacity. In this study, we present findings from a secondary analysis of a qualitative interviews with physicians. These interviews were initially used to assess usability of an instrument for the evaluation of decision-making capacity. By looking at difficult cases of decision-making capacity evaluation in patients with dementia, we provide recommendations for such evaluations in clinical practice.

**Methods:**

We used thematic coding to analyse physicians’ narratives of problematic decision-making capacity evaluations in patients with dementia to uncover challenging issues of decision-making capacity evaluation.

**Results:**

In this study, decision-making capacity evaluations in patients with dementia were mainly perceived as challenging when they pertained to treatment refusals and treatment unrelated circumstances, such as psychiatric consultation, advance directives, and new living arrangements. Furthermore, the physicians reported training needs regarding situation-independent challenges with decision-making capacity evaluation.

**Conclusions:**

Upon further examining self-reported training needs and challenging cases, we have developed recommendations to improve decision-making capacity evaluations in clinical practice. In these recommendations, we argue that being able to evaluate decision-making capacity is an integral part of the informed consent process.

## Background

Physicians have a legal and moral duty to obtain informed consent from their patients for medical treatment [1]. For a consent to be legally valid, the patient must be informed thoroughly, make a voluntary choice, and have decision-making capacity (henceforth DMC [2]). As a prerequisite to valid consent, DMC has become an indispensable pillar of modern patient-centred medicine. However, the clinical evaluation of DMC is difficult, especially in patients with dementia [3–5]. In dementia, cognitive impairments often limit the ability to understand, retain, and weigh different treatment options [6, 7]. For example, cognitive fluctuations are a recognised challenge to DMC evaluation [8]. Although cognitive fluctuations occur more often in cases of dementia with Lewy bodies, where it is a distinct diagnostic criterion, fluctuations in attention and memory are widely prevalent in all forms of dementia [9, 10]. Nevertheless, it has been acknowledged that being diagnosed with dementia does not render a patient incompetent per se [11–13].

Dementia is a common condition in the elderly population, and in light of demographic changes, it is of particular clinical importance. The World Alzheimer Report [14] estimated that the number of people affected by dementia will double every 20 years, that is, by 2040 over 80 million people will be affected by dementia [15]. As a result, DMC evaluation of patients with dementia will become an even more frequent concern for physicians. In light of this, it is important that the physicians are properly trained to evaluate DMC. However, a recent scoping review of the literature found that training of physicians on DMC and its evaluation is suboptimal [16]. In focus group interviews with physicians, the same study delved into major barriers with regard to the evaluation of DMC, including, in particular, inconsistency in approaches to DMC evaluation, and lack of knowledge about the potential ethical tension between patient autonomy and patient protection.

While theoretical aspects of DMC and instruments/tools for its evaluation have been widely researched (e.g. [17–19]), studies focusing on the actual DMC evaluation in clinical practice are rare [20].

### Aim of the study

In the present study, we explored how physicians actually carry out DMC evaluations and what issues they perceive as challenging. In a secondary analysis of qualitative data from an interview study with physicians, we extracted difficult cases of DMC evaluations with patients with dementia from physicians’ everyday practice as well as the physicians’ self-reported training needs. This provided the basis for the discussion of challenging feature and general recommendations for physicians.

## Methods

The present study is based on qualitative interviews which were part of a nationwide research project on DMC in Switzerland funded by the Swiss National Science Foundation (SNSF) (for details, see [21]). As part of this project, an evaluation instrument was developed to account not only for abilities of the patient, but to also capture physician-associated factors (e.g., intuitions, value judgements) in the decision-making process (for details, see [22–24]). The interviews were part of an evaluation of usability and feasibility of this instrument. As confirmed by the cantonal ethics commission of Zurich, Switzerland, in January 2015 in accordance with the Swiss Human Research Act, ethical review by the cantonal ethics commission was not required because no health-related personal data from the assessed physicians has been gathered in the present study. Written informed consent was not necessary. However, all participants consented verbally to participate.

### Participants

A total of 24 healthcare professionals took part in the project of which 18 study participants were physicians. These initial participants were sampled by convenience. For the present study, we focused on the perspectives of the physicians as they are often legally responsible for the evaluation of DMC as attending physicians. We excluded the five nurses and one psychologist from the analysis. The physicians worked in internal medicine (*n* = 10), in psychiatry or psychiatric consultation (*n* = 6), or in other specialties (*n* = 2).

### Qualitative data

All study participants received two-hour training on the concept and evaluation of DMC, for which they received six credits by the Swiss Institute for Advanced Medical Training and Education. In the subsequent four-month period, participants were asked if evaluation of DMC was an issue in their clinical practice, to use the newly developed DMC evaluation instrument to evaluate the patient’s DMC. After 4 months, face-to-face semi-structured interviews were conducted (see [21]). The main aim of the interviews was to assess qualitatively the feasibility and usability of the instrument in everyday clinical practice. Study participants were also asked about difficulties in evaluating DMC and about their perceived training needs in the evaluation of DMC. Furthermore, participants were asked to report cases from their experience during the last 4 months where the patient’s DMC was questionable. The interview language was Swiss German and interviews were conducted between April and November 2016. All interviews took place at the workplace of the study participant (e.g. hospital, general practice), during which only the interviewer and the interviewee were present. The mean length of the interviews was 45 min. Interviews were audio recorded and transcribed verbatim into written German.

### Data analysis

The secondary analysis of the interview data firstly focused descriptively on the difficulties study participants reported when evaluating DMC in patients with dementia. To be within this category, it was necessary that (a) the patients who were discussed by the participants had a diagnosis of dementia, and (b) that the physician participants reported that they had significant problems in evaluating DMC in these patients. Of the 26 patient cases discussed by the 18 participants, 9 physicians discussed 11 cases fulfilling these conditions. Secondly, the answers of the same study participants with regard to their own training needs were aggregated (5 out of 9). Thirdly, the resulting data set for this paper from the above two procedures was inductively analysed with thematic analysis [25] to further understand the needs in dementia-related DMC evaluation.

One author (CP) carried out the data extractions and aggregations. All co-authors read the extracted data and discussed ethical and medical themes inherent in the physicians’ case narratives, which were grouped into three prevailing themes: (1) treatment refusals, (2) treatment unrelated circumstances, and (3) situation-independent challenges of DMC evaluation.

## Findings

In this section, the clinical situations corresponding to the themes and which made DMC evaluation challenging are presented. In the next section, the ethically and medically challenging issues inherent in the aforementioned evaluation of DMC in patients with dementia are discussed. Moreover, we provide recommendations for challenging issues in the evaluation of DMC.

### Clinical situations that challenge evaluation of DMC

#### Theme 1: when patients with dementia refuse treatment

In three of the reported challenging cases (cases 1, 6, and 8), participants described patients who refused treatment even though severe medical risk was involved in not having the treatment. In all cases, the refusal was respected by the responsible physician despite the absence of the patient’s DMC.

Case 1: An elderly patient with severe dementia refused to undergo surgery for her malignant tumour. In addition, her dementia had deteriorated before refusing to undergo surgery. The tumour was likely to grow and there was a high risk of tumour related infection. In earlier oral statements, she had also expressed her refusal of surgery. The attending physicians deemed her to lack DMC to refuse treatment. However, her daughter was present and stressed the importance to respect her wish. In the end, the physicians did not conduct the surgery.

Case 6: A patient of old age with mild dementia refused follow-up chemotherapy after tumour surgery. The physician had to inform the patient repeatedly about the risks and benefits as she had forgotten the relevant information after each appointment. The patient’s reason for deciding against the chemotherapy was that it would lead to hair loss. At each appointment, the decision not to undergo chemotherapy was consistent and she was deemed capable of decision-making.

Case 8: An elderly patient refused a surgical amputation of his gangrenous leg. As it is discussed further below, the patient underwent psychiatric consultation and was evaluated to lack DMC. In this case, there was no family member present, nor were there any advance oral statements or written directives and the refusal implied severe medical risk including death from septicaemia. Nonetheless, the physicians respected the patient’s wish which led to his death.

#### Theme 2: treatment-unrelated circumstances

DMC evaluations were not only found to be complicated by apparently risky treatment refusal but were reported as difficult by the study participants in several situations such as when psychiatrists had to be involved in the DMC evaluation process, DMC in the context of advance directives (AD), and DMC when patients wanted to decide further care or living arrangements.

##### Psychiatric consultation

In two of our cases (cases 2 and 8), the attending physician asked for psychiatric consultation to assist with DMC evaluation. In both cases the attending physician did not formally evaluate DMC and the psychiatrist declared the patient not to have DMC to make decisions. Despite this conclusion from the psychiatrist, the attending physician did not recommend the patient to undergo the indicated treatment procedure, notwithstanding apparently negative consequences for the patient if they did not have the treatment (i.e., death of the patient in case 8).

##### Advance directives

When writing an advance directive, DMC has to be evaluated if there is reasonable doubt that the patient might lack DMC as the advance directive is only valid if developed by a competent patient. Nevertheless, in most legal systems every adult person is presumed to have DMC (e.g., Swiss Civil Code 2018, Art. 16; UK: Mental Capacity Act 2005, section 1, 2nd principle). In Switzerland, advance directives are binding by law, but need not satisfy specific formal requirements. In advance directives, a legal guardian can be appointed. If there is no advance directive, there is cascade of guardianship among the closest family members and relatives.

In case 4, the patient was deemed unable to write an advance directive as he did not have the DMC to understand what was discussed. In this case, legal authorities became involved and a family member was appointed as his guardian. In case 10, the physician had doubts about the patient’s DMC, but the advance directive was still written due to the presence of the family who confirmed the patient’s wishes which helped the physician to conclude that the patient was competent (no further details could be extracted from the data). In case 3, a patient with moderate dementia had prepared an advance directive with a notary, in which her son was appointed as the primary guardian. She then accused her son of stealing her money. The physician could neither evaluate DMC retrospectively nor decide whether the patient had been capable of writing the advance directive. The physician could not certify the irrationality of the assertion that the patient’s son was stealing. The case was subsequently transferred to the respective legal authorities (i.e. child and adult protective services).

##### New living arrangements

Furthermore, DMC is often in question when new living and care arrangements for elderly patients with dementia are discussed. In case 5, an older man with dementia who had a wakened night shift wanted to leave the hospital. The physicians doubted whether he was able to care for himself at home. Two cases (cases 7 and 9) deal with patients with dementia whose circumstances were such that family members or healthcare professionals who were unable to provide the needed care at home and urged these patients to move into nursing homes. In these cases, physicians either let the patient go home under surveillance (case 5), persuaded or informed the patients repeatedly about the need to go into a nursing home (both case 7 and 9).

#### Theme 3: situation-independent challenges in decision-making capacity evaluation

Several of the study participants reported having had difficulties with different legal issues related to DMC evaluation. One study participant reflected:

So, I wouldn’t have known for example, or at least I wouldn’t have known it directly, that this [lack of DMC] is not required when ordering involuntary admission^1^ (mhm), hence I believe that there could be a lack of information. – Interviewee 17 (case 9).

Not knowing involuntary admission is independent of a person’s DMC is an example of lack of legal knowledge in the context of DMC evaluations, but this is not specific to dementia. For example, physicians found it difficult to evaluate DMC when the patient was subject to a guardianship order or had instated a lasting power of attorney. One participant noted that in her, falsely held, view the law might contradict itself in these cases:

I question if it is necessary for a full guardianship that the patient does not have DMC. Children and adult protection law formulate it this way.^2^ This bothers me. DMC is always dependent on a certain situation and you cannot generally declare someone as not having DMC per se and put a guardianship in place. — Interviewee 2 (case 2).

Apart from uncertainty about the law with regard to DMC, the clinical evaluation of DMC was a concern for the study participants. However, a variety of instruments exist to evaluate DMC, e.g. the MacCAT-T [26]. One physician expressed the wish for accessible screening instruments in DMC evaluations:

So, I think that would be a good thing, I mean, we now work a lot with scores and such on the derma [dermatological station] to assess the severity and it is easy, and you can get training then […] sure, that training [on DMC] would be more complex and one would have to train with cases, as we did in the training session. – Interviewee 8 (case 7).

In reports about clinical evaluation of DMC, several study participants emphasised the need for economical, reliable screening instruments. Another physician stated that

it would be wonderful if there were any questionnaire with scores. I am working in assessment. I am asking 10 questions. If the patient has a score of 4, he has a depression. It would be nice if this existed [for DMC]. But I, too, don’t see how. – Interviewee 1 (case 1).

In general, the role cognitive screening instruments play in DMC evaluation is reported to be unclear, for example, whether a cut-off score can be an indicator of lack of DMC. In one case (case 4), cognitive testing alone was used to evaluate DMC.

## Discussion: challenging issues raised when evaluating decision-making capacity of patients with dementia

### A patient with dementia refuses treatment

In all clinical cases, treatment refusal was respected. No patient was forced into undergoing treatment. In practice, there could be a different threshold applied for refusal than for consent to treatment. Furthermore, consequences of not respecting refusal might also be graver than respecting it. This is in line with the legal “right to refuse” life-prolonging treatment [27] of competent patients. However, a prerequisite for the refusal is that the patient is able to make a considered decision. When the patient lacks DMC, physicians are morally obliged to promote the welfare of their patients. In Table 1, challenging issues of the cases are presented in which informed refusal was complicated by dementia.

**Table 1:** Treatment refusal in cases of dementia

Refusal	Challenging issue
Minor intervention with a low or unclear risk involved where the patient has no living relatives or legal representatives.	Mild forgetfulness: Information about the intervention is lost after each appointment, but the attention and awareness are preserved so that momentary informed consent is possible.
Intervention for major, but non-life-threatening disease.	Severe cognitive deficits, but earlier oral statements consistent with the refusal.
Life-saving surgery (e.g., necessary amputation) and the patient has no living relatives or next of kin.	Patient lacks capacity according to the psychiatric consultant, but to neglect the refusal would mean to heavily infringe on the patient’s actual expressed will.

### A patient with dementia wants to write an advance directive

The question of DMC arises in the context of advance directives at different times. First of all, DMC is needed at the time of writing an advance directive as well as when changing it. Once a legally valid advance directive is in place, it can be open to challenge in court if there is reasonable doubt that the patient lacked DMC at the time of writing the advance directive. In Table 2, challenging issues for DMC evaluations from the cases of the present study are presented.

**Table 2:** Issues with advance directives

Status of advance directive	Challenging issue
The patient has set up an advance directive already. The patient now wants to change the primary care taker enlisted in the advance directive.	Capacity is in doubt and no thorough formal evaluation at the time of the writing of the advance directive is available.
The patient wants to set up an advance directive in the presence of the family.	The presence of and support for the advance directive by the family might suggest that a formal capacity evaluation is not required.

This result seems to emphasize the importance of advance care planning (ACP) in dementia, for example through general practitioners [28]. However, ACP in dementia is also challenging [29]. Indeed, the process of ACP in dementia shares the general challenges of evaluation of DMC outlined in this article, for example the involvement of family caregivers and relatives [30].

Closely related to advance directives is the question about what to do when a patient cannot be supported and cared for at home any longer. Similarly, as with advance directives, the role of the family and DMC evaluation presents a challenging issue.

### A patient is unable to care for himself but wants to go home

Care decisions are treatment unrelated but nonetheless can considered as health decisions. As such, DMC is often evaluated to decide upon alternative care arrangements when the needed care cannot be provided by informal caregivers at home or outpatient nursing teams (see Table 3). These cases often need a multi-professional team effort, involving physicians and social workers alike.

**Table 3:** Capacity when care cannot be provided

Care situation	Challenging issue
The patient is admitted to the hospital because care cannot be provided at home.	The patient is unwilling to resettle into a nursing home.
The patient is unable to care for herself at home and was hospitalized several times before.	The patient wants to stay at home.

Physicians encounter these challenging issues by finding a solution to minimise risk and maximise medical benefit. This has often little to do with a formal DMC approach. Physicians negotiate and try to get acceptance of patients for their transfer to a nursing home.

## Conclusion: recommendations to address decision-making capacity evaluations of patients with dementia

### Evaluation of decision-making capacity as part of the informed consent process

We recommend DMC evaluation as part of a nuanced informed consent or informed refusal process [31]. A formal DMC evaluation becomes necessary if there is reasonable doubt that the patient might lack DMC, e.g. in the case of a patient with a diagnosis of dementia. Focussing on informed consent ensures that first all reasonable steps have to be taken to provide the necessary information for the patient to decide. For example, the importance of informing the patient in a thorough and easy comprehensible manner should not be underestimated [1].

Furthermore, the physician should assess and ask the family when and where the patient is cognitively at their best, e.g. if the patient experiences confusion later in the day (‘sundowning’). If there is temporary fluctuation in attention and memory [8] the informed consent process should be repeated at least two different times before a final decision is made [32]. If sufficient information is provided and the patient still seems unable to coherently consent or refuse treatment, the physician’s clinical judgement is needed about whether or not the patient has the DMC to make decisions.

We agree that this judgement is subjective [11, 22] in the sense that the physician’s values, observations, and own experiences may influence it. It is suggested that physicians discuss these cases in case conferences and supervision sessions. This clinical judgement can also be informed by a psychiatric consultation in some cases.

### Psychiatric evaluation of decision-making capacity

It has been called a myth of DMC that only a psychiatrist can evaluate DMC [33]. In cases where dementia is related to, or concomitantly present together with, severe mental disorder, psychiatric consultation is advisable. Nevertheless, DMC evaluations by psychiatric consultants are not overruling the clinical judgement of the treating physician, who retains legal responsibility for the evaluation. In addition, the attending physician (in general) is most informed of risks and benefits of the decision at hand, has known the patient the longest, and can judge best the state the patient is in. In dementia, the cognitive state of patients can also be assessed by neurologists and neuropsychologists to inform the DMC evaluation of the treating physician. If psychometric instruments such as the Mini-Mental-State-Exam (MMSE [34]) are employed, these should only be used as a decision-aid and not as stand-alone diagnostic tool for the patient’s DMC. Even the use of low cut-off scores, as is use for consent to research less than 10 of 30 in the MMSE [35], runs the risk of neglecting patients with dementia, who might have otherwise decided for themselves. Although psychometric instruments have an important place in practice, they cannot substitute clinical judgement. This clinical judgement can be supported by tools focusing on the process of DMC evaluation (e.g. from the Swiss context, the U-Doc [24]).

### Solution-focused decision-making while respecting the right to autonomy

The use of solution-focused approaches if DMC is lacking is common in multifaceted medical decisions. We understand solution-focused approaches akin to a casuistic approach to decision-making based on human rights [36]. This human rights approach is different to a mental capacity/welfare-based approach because human rights such as outlined in Article 5 and 8 ECHR are independent of DMC evaluations. One example might be that “the detention of a person lacking DMC cannot be justified simply on the basis that doing so is in their best interests” [36].

Another example closer to our cases, a physician might let a patient go home even though he considers him incapable of decision-making. He might ask the patient to report back or ask family members to report about the state of the patient’s health. This way, the patient’s right of autonomy is preserved but the physician’s corresponding duty to protect the patient is served also.

Unclear at this point is what constitutes valid solution-focused approaches based on human rights without involving legal institutions. One key aspect might be good patient-physician communication, which can convince the patient of the need for further care without being paternalistic or controlling, hence infringing on the right to autonomy [37]. Furthermore, in cases where the appointment of a guardian is not possible, it might become necessary to involve social workers and the responsible legal institutions, e.g. child and adult protective services in the Swiss context. Using a right-based approach, the legal institutions, e.g., will “consider the issue from the perspective of each relevant party, explain their rights and justify, where appropriate, the interference in their rights” [36]. While solution-focused approaches do not rely solely on formal DMC evaluations, in practice this kind of decision making is of utmost importance.

### Presence of relatives and the evaluation of decision-making capacity

If the physician concludes that the patient cannot consent, legal guardianship by family members or other relatives is advisable. A right-based approach has the unique advantage that it can balance conflicts between family members and, as already pointed out, does not simply overrule patient autonomy.

It is suggested that the guardianship or lasting power of attorney is appointed by, for example a court of protection. This legal recognition serves as a safeguard for family members. Furthermore, notwithstanding that the patient lacks DMC, the patient’s preferences and wishes should determine decision making as he still has a right to autonomy. In establishing patients’ preferences, family members can also serve as informants.

## Conclusion

Medical and ethical aspects of right-based approaches in clinical practice are a neglected field in decision-making research. The involvement of family and relatives as guardians demand closer attention in the context of dementia. Further research should examine solution-focused decision making in a clinical context.

## Data Availability

The datasets analysed during the current study are available from the corresponding author on reasonable request.

## Abbreviations

AD: Advance Directives
MacCAT-T: MacArthur Competence Assessment Tool-Treatment
MMSE: Mini-Mental-State-Exam
SNSF: Swiss National Science Foundation

## Footnotes

1 In Switzerland, under Art. 426 of the Civil Code, involuntary admission is possible independent of decision-making capacity. However, involuntary treatment of competent persons is not allowed.

2 This is actually not the case in Switzerland. Permanent absence of capacity will or could lead to full guardianship – the unofficial Swiss translation uses General Deputyship, but full guardianship does not necessarily mean permanent absence of capacity under Art. 398 of the Swiss Civil Code.
